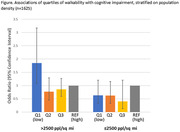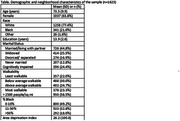# Associations of Neighborhood Walkability with Cognitive Impairment Depend on Geographic Context

**DOI:** 10.1002/alz.089103

**Published:** 2025-01-09

**Authors:** Andrea L Rosso, C. Elizabeth Shaaban, Greta Cheng, Jeanine M Buchanich, Meryl A Butters, Annie Cohen, Tamara Dubowitz, Melanie Y Faulkner, Marissa A. Gogniat, Oscar L. Lopez, Anum Saeed, Steven E. Reis, Wendy M Troxel, Andrea M. Weinstein

**Affiliations:** ^1^ University of Pittsburgh, Pittsburgh, PA USA; ^2^ University of Pittsburgh Alzheimer’s Disease Research Center (ADRC), Pittsburgh, PA USA; ^3^ University of Pittsburgh School of Medicine, Pittsburgh, PA USA; ^4^ RAND Corporation, Pittsburgh, PA USA; ^5^ University of Pittsburgh Alzheimer’s Disease Research Center, Pittsburgh, PA USA

## Abstract

**Background:**

Neighborhood walkability may affect cognitive impairment through promotion of physical activity. However, most studies are conducted in urban, predominantly White samples. We assessed how walkability is related to presence of cognitive impairment and whether the relation differs by neighborhood population density (differences in likelihood of promoting physical activity) and/or racial composition (differences in quality of neighborhood resources). We hypothesized that lower walkability is related to greater likelihood of having cognitive impairment and that this association is stronger in areas with higher population density (i.e., more urban) and with a lower percentage of Black residents.

**Methods:**

We used cross‐sectional data from 4 harmonized studies of cognition and dementia across Southwestern Pennsylvania (n = 1625, Table). Walkability was defined by quartiles of the Environmental Protection Agency walkability index at the participant’s residential census tract. Cognitive impairment was defined as clinically adjudicated mild cognitive impairment or dementia (3 studies) or a Clinical Dementia Rating of ≥0.5 (1 study). US census data were used for population density (> or ≤ 2500 people/square mile) and racial composition (0%‐10%, >10%‐50%, or >50% Black residents) of each census tract. Logistic regression models with robust standard errors to account for clustering at the census tract modelled the association of walkability and cognitive impairment with adjustment for participant age, sex, years of education, marital status, race, and tract area deprivation index. Multiplicative terms were included in the models to test interactions of walkability with either population density or racial composition. Models were stratified when interaction p‐values <0.1.

**Results:**

In adjusted analyses, walkability was not related to cognitive impairment in the full sample (OR = 0.89 (95% CI: 0.61‐1.28 for highest vs lowest quartile). Walkability interacted with population density (p interaction = 0.09 for highest vs lowest quartile), such that low walkability was associated with greater likelihood of cognitive impairment, but only in areas with high population density (OR = 1.85 (95% CI: 1.08‐3.17 for highest vs lowest quartile; Figure). There was no interaction with racial composition.

**Conclusions:**

Neighborhood walkability may be an important population‐level modifiable risk factor for cognitive impairment but accounting for geographic context such as urbanicity is important for considering implications and interventions.